# The Irish Nurses' Tribute Fund

**Published:** 1918-11-09

**Authors:** 


### THE EDITOR'S LETTER-BOX.

**[Correspondence on all subjects is invited, but we cannot in any way be responsible for the opinions expressed by our correspondents, who must give their name and address as a guarantee of good faith, but not necessarily for publication. Correspondents are reminded that brevity of style and conciseness of statement greatly facilitate early insertion.]**

### The Irish Nurses' Tribute Fund.

*To the Editor of* THE HOSPITAL.

SIR,—Permit me to tell a little story.

A few months ago Sir Arthur Stanley paid a visit to Ireland in order to inaugurate the Nurses' Tribute Fund in that country. He was the guest of Lady Arnott during his brief stay, and, as everybody in Ireland knows, Lady Arnott is very influential, of exceptional ability, and enthusiastic, as well as successful in her undertakings. She entered with zest into the Tribute Fund movement, gave a handsome subscription of several hundred pounds, formed a committee, and the result of the whole is that £10,000 has been realised. So far so good. But there is another side. The College of Nursing and the Tribute Fund to Nurses are Sir Arthur Stanley's children, and, since they are not the offspring of Mrs. Bedford Fenwick, this lady's Irish satellites hatched a conspiracy to kidnap the Tribute idea, and, as far as possible, to dissociate the movement from its legitimate parent. With this object in view, they adopted the Sinn Fein motto, "Ourselves alone," preached the usual clap-trap about distrust of England (although the women who did this are English and Scottish), and thus prevented Irish nurses from entering into the great sisterhood of British nurses without paying them the compliment of consulting their wishes or giving them any opportunity of self-determination.

Four "commissioners" are now to be appointed to administer this £10,000 Fund, and the following interesting questions arise—Who are to be the commissioners? Who are to be the beneficiaries? What figure, if any, are Sir Arthur Stanley and the College of Nursing to cut in the matter? Who is to determine what is a "trained nurse"?—for none but such is eligible to participate.

Nursing in Ireland at the present time is in a somewhat chaotic condition, utterly unprogressive, and without co-operation or cohesion. The Bedford-Fenwick Irish school are without any policy save that of maintaining their own oligarchy and of damaging the College which seeks to gather into its fold all the trained nurses, not only of the kingdom, but of the Empire.

Now it is desirable to point out that this £10,000 tribute owes its entire success to the fact that Sir Arthur Stanley enlisted the sympathy of Lady Arnott. Of this there is no room for doubt. The opposition clique did absolutely nothing except the destructive and disreputable work of keeping the College and its worthy President in the background. Not a shilling did they influence, and not a shilling could they influence. They are pirates, pure and undiluted. If you want proof of this, see the Bedford-Fenwick organ, current issue, where "an impudent gamble," being a Stock Exchange lottery, is held up to derision as an "insult" to the profession.

May I ask Mrs. Fenwick, or her Irish school, one simple question? What have they, jointly and generally, or individually, ever in their thirty years of "leading" done for the financial betterment of any nurse, Irish or English? Have they done aught but screech? How much of the £10,000 can they earmark as their effort? If Lady Arnott had not mothered the movement, where would it be now?

But mark you, Sir, now that it is all gathered in, they want to do two things. One is to cavil at the methods of collection, and the other is to administer the Fund.

I hold the view that the Irish Nurses' Tribute is the child of the College of Nursing, and of Sir Arthur Stanley. The Fund is very badly needed. There are hundreds of nurses, trained women, many of gentle birth, in absolute poverty in Ireland at the present time—women who have fulfilled their duty and who are too old or too infirm to labour further, and to whom half-a-crown is a godsend. This I can prove, and I can likewise prove that this school of pirates and high-toned critics, whose efforts consist of screeching, have never in the whole course of their existence raised a finger to help the hard-up section of the profession. They do not either know or want to know their names.

Mark my words, "Wait and see," and you will find that they will screech very loud if they are denied the privilege of distributing the "insults." It is up to the College to see that the Irish Tribute is placed to its credit. Sir Arthur Stanley, the nurses' champion, created this Fund. Without him it would never have been thought of. Without Lady Arnott it would never have been collected. I suggest that Sir Arthur Stanley and Lady Arnott should appoint the commissioners who are to administer the Fund, and in making this suggestion I am interested only in the welfare of the unrepresented, silent, forgotten, and poverty-stricken Irish nurse.

A CONTRIBUTOR TO THE FUND.

